# 2408: Sleep apnea is associated with increased risk for sudden unexpected death in epilepsy (SUDEP)

**DOI:** 10.1017/cts.2017.105

**Published:** 2018-05-10

**Authors:** Anwar A. Chahal, Thao Luu, Paul Timm, Paul Sheppard, David Sandness, Ashley Enke, Lucas Dueffer, Max Dresow, Stuart J. McCarter, Virend K. Somers, Erik K. St. Louis

**Affiliations:** Mayo Clinic, Rochester, MN, USA

## Abstract

OBJECTIVES/SPECIFIC AIMS: To assess the association between probable OSA and the sudden unexpected death in epilepsy (SUDEP-7) risk profiling index in monitored adult inpatients with epilepsy. METHODS/STUDY POPULATION: We analyzed 49 consecutive adults (>18 years) with refractory epilepsy admitted to our inpatient epilepsy monitoring unit. The SUDEP-7 inventory was performed for all subjects. Probable OSA was identified using overnight oximetry, the Sleep Apnea Sleep Disorder Questionnaire (SA-SDQ), and STOP-BANG inventory. RESULTS/ANTICIPATED RESULTS: Thirty-nine percent of participants screened positive for probable sleep apnea. Patients with high SUDEP-7 scores were more likely to have a positive screen for OSA. DISCUSSION/SIGNIFICANCE OF IMPACT: OSA is an independent risk factor for sudden cardiac death. OSA may be a hitherto unrecognized contributor to sudden death risk in epilepsy. Further studies determining the relationship between OSA, neural circulatory control and SUDEP are warranted.

